# Associated Factors for Falls among the Community-Dwelling Older People Assessed by Annual Geriatric Health Examinations

**DOI:** 10.1371/journal.pone.0018976

**Published:** 2011-04-19

**Authors:** Chung-Hao Lin, Kuo-Chen Liao, Shou-Jin Pu, Yung-Chang Chen, Maw-Sen Liu

**Affiliations:** 1 Department of Internal Medicine, Chang Gung Memorial Hospital, Taipei, Taoyuan, Taiwan; 2 Chang Gung University College of Medicine, Taoyuan, Taiwan; Yale University School of Medicine, United States of America

## Abstract

**Background:**

Falls are very common among the older people. Nearly one-third older people living in a community fall each year. However, few studies have examined factors associated with falls in a community-dwelling population of older Taiwanese adults.

**Objectives:**

To identify the associated factors for falls during the previous 12 months among the community-dwelling Taiwanese older people receiving annual geriatric health examinations.

**Participants:**

People aged sixty-five years or older, living in the community, assessed by annual geriatric health examinations

**Methods:**

1377 community-dwellers aged ≥65 years who received annual geriatric health examinations at one hospital in northern Taiwan between March and November of 2008. They were asked about their history of falls during the year prior to their most recent health examination.

**Results:**

The average age of the 1377 participants was 74.9±6.8 years, 48.9% of which were women. Three-hundred and thirteen of the participants (22.7%) had at least one fall during the previous year. Multivariate analysis showed that odds ratio for the risk of falling was 1.94 (95% CI 1.36-2.76) when the female gender group is compared with the male gender group. The adjusted odds ratios of age and waist circumference were 1.03 (95% CI 1.00–1.06) and 1.03 (95% CI 1.01–1.05) respectively. The adjusted odds ratios of visual acuity, Karnofsky scale, and serum albumin level were 0.34 (95% CI 0.15–0.76), 0.94 (95% CI 0.89–0.98), and 0.37 (95% CI 0.18–0.76) respectively. Larger waist circumference, older age, female gender, poorer visual acuity, lower score on the Karnofsky Performance Scale, and lower serum albumin level were the independent associated factors for falls.

**Conclusion:**

In addition to other associated factors, waist circumference should be included as a novel risk factor for falls.

## Introduction

Falls are very common among the older people. Nearly one-third older people living in a community fall each year. Fall rates continue to increase with age [1]–[3]. According to the Taiwan National Health Interview Survey in 2005, the prevalence of falls was 20.5%, which was similar as previous studies in Singapore[4], Hong Kong [5], and China [6]. Although most falls produce no serious injury, 5–15% of the community-dwelling fallers suffer from serious injuries, such as fractures, serious lacerations, head injuries, and even death [5]. Fall-related injuries require medical treatment including hospitalization and considerable healthcare expenditure. Falls in older people can also result in disability, fear of falling, reduced quality of life, loss of independence, and institutionalization [7].

The risk factors for falls include co-morbidities, cognitive impairment, neuromuscular impairment, balance and gait disorder, depression, functional decline, higher use of medication, and environmental hazards [8]. Frequently, community-dwelling older people neither recognize the risk factors for falls nor report their falls to their physicians [9]. The risk factors for falls only become evident after injuries and disability have occurred. Therefore, assessment of the associated factors for falls in annual geriatric health examinations is prerequisite as regard to fall prevention [10]. The objective of this study was to identify possible associated factors identified during an annual geriatric health examination among a Taiwanese population.

## Methods

### Study design

This is a cross-sectional study according to the health examination program opened to senior citizens in urban area who are aged 65 years or older.

### Patient information and data collection

Participants in the study were 1377 community-dwellers aged 65 years and older who received annual geriatric health examinations at one hospital in northern Taiwan between March and November 2008. Most participants signed up for the health examination program were independent community-dwelling older adults. Participants were excluded from the study if they could not confirm any information about falls. This study was approved by the institutional review board at Chang Gung Memorial Hospital. The written informed consents were obtained from all participants involved in the study. Physicians identified fallers by using the standard definition of a fall.

### Definition

Information about falls was self-reported from participants. A fall is defined as an event that results in a person coming to rest unintentionally on the ground or another lower level, not due to any intentional movement, major intrinsic event, or extrinsic force. Participants were uniformly asked about their history of falls during the year prior to their most recent health examination. The recall period is one year. The recall bias would impact the finding and lower the prevalence of falls.

### Documentation

As part of our annual geriatric health examination, the co-morbidities were documented. Body height, weight, body mass index, systolic and diastolic pressure, and waist circumference were measured. Waist was measured at the level midway between the lateral lower rib margin and the superior anterior iliac crest. Visual acuity was assessed with a Snellen chart placed at a distance of 6 m. The information about co-morbidities, such as hypertension, Diabetes mellitus, Parkinson's disease, dementia, or arrhythmia was from the patient's statement on the record of health examination. Overall functional and physical performances were determined by the Karnofsky Performance Scale. The Karnofsky Performance scale was an effective score for the functional status of geriatric patients. It is uncomplicated and easy to evaluate abilities to carry on normal activity and to work, care for most personal needs with varying amount of assistance needed, potential disability with institutional or hospital care, and progression of disease [11]. Other measures we used in the geriatric examination included the Short Portable Mental Status Questionnaire (SPMSQ) for cognition and the 5-item Brief Symptom Rating Scale (BSRS) to identify possible depression and anxiety. Three or more errors of SPMSQ would identify the person as impaired cognition [12]. When administered to community-dwelling older adults, the specificity is found to be better than 90%. According to the previous study, the internal consistency coefficient for the BSRS-5 was 0.84 and area under the receiver-operating characteristic curve was 0.91. The optimal cut-off point was 5/6. The BSRS-5 is an effective screening instrument for the identification of psychiatric morbidity in hospital-based health screening settings [13]. A complete blood count (CBC) examination was performed, and fasting blood glucose, cholesterol, triglyceride, aspartate transaminase (AST), alanine transaminase (ALT), blood urea nitrogen (BUN), creatinine, and albumin levels were measured.

### Statistical analysis

The statistical analysis was performed using SPSS 12.0 for Windows. Descriptive statistics are expressed as mean±SE. The Student's t-test was used to compare the means of continuous variables and normal distribution data in associated factors for falls. Categorical data were tested using χ^2^ analysis. A multivariate analysis was performed by applying a multiple logistic stepwise regression procedure to obtain variables that independently correlated with falls. All statistical tests were two-tailed, and a significance level of p = 0.05 or less was used.

## Results

The average age of the 1377 participants was 74.9±6.8 years, and 48.9% of which were women. As assessed by annual geriatric health examinations, 313 of the participants (22.7%) had experienced at least one fall during the previous year. The characteristics that differentiate fallers from non-fallers are shown in Table 1. Falls were more common among the older people and among women, as 27.3% of women and 18.3% of men experienced falls during the previous year. There was no difference in co-morbidities such as hypertension and diabetes mellitus between fallers and non-fallers. Participants with Parkinson's disease, cardiac arrhythmia, and dementia had a higher risk of falling.

**Table 1:** Characteristics of faller and non-faller (n = 1377).

	Total	Non-faller (n = 1064)	Faller (n = 313)	p-value
Gender (female)	673 (48.9%)	489 (45.9%)	184 (58.8%)	<0.001
Age (year)	74.9±6.8	74.4±6.5	76.6±7.2	<0.001
Hypertension	516 (37.5%)	402 (37.8%)	114 (36.4%)	0.69
Diabetes mellitus	150 (10.9%)	114 (10.7%)	36 (11.5%)	0.68
Parkinson's disease	17 (1.2%)	8 (0.8%)	9 (2.9%)	0.006
Dementia	9 (0.7%)	4 (0.4%)	5 (1.6%)	0.03
Arrhythmia	24 (1.7%)	10 (0.9%)	14 (4.5%)	<0.001
BSRS-5 ≧6	244 (17.7%)	169 (15.9%)	75 (24.0%)	0.001
SPMSQ ≧3	29 (2.1%)	15 (1.4%)	14 (4.5%)	0.003
Karnofsky Scale	89.4±3.8	89.9±3.1	88.3±5.0	<0.001
Visual acuity	0.36±0.23	0.37±0.24	0.32±0.21	<0.001
Body weight (kg)	61.4±10.2	61.7±10.1	60.5±10.3	0.07
Height (cm)	158.6±8.4	159.1±8.2	156.8±8.7	<0.001
BMI (kg/m^2^)	24.4±3.3	24.3±3.3	24.6±3.5	0.16
Waist circumference (cm)	87.1±9.5	86.7±9.2	88.5±10.4	0.004
SBP (mmHg)	130.3±17.6	130.1±17.5	130.9±18.1	0.53
DBP (mmHg)	73.1±24.7	73.5±27.4	71.6±10.7	0.23
Glucose (mg/dL)	103.0±23.2	102.6±22.9	104.2±24.4	0.34
Hemoglobin (g/dL)	13.8±1.4	13.9±1.4	13.5±1.4	0.001
Albumin (g/dL)	4.5±0.3	4.5±0.3	4.4±0.3	<0.001
AST (U/L)	25.1±16.6	25.1±15.3	25.3±22.8	0.92
ALT (U/L)	24.1±24.8	24.3±26.6	23.4±16.8	0.62
BUN (mg/dL)	16.7±6.1	16.5±5.7	17.3±7.5	0.07
Creatinine (mg/dL)	0.97±0.50	0.96±0.36	1.03±0.81	0.02

Data expressed as mean±SD for continuous variables in normal distribution.

Mean [median value] for continuous variables not in normal distribution.

All statistical tests were two-tailed, and a significance level of p = 0.05 or less was used.

SPMSQ, the Short Portable Mental Status Questionnaire; BSRS-5, the 5-item Brief Symptom Rating Scale; BMI, body mass index; SBP, systolic blood pressure; DBP, diastolic blood pressure; AST, aspartate aminotransferase; ALT, alanine aminotransferase; BUN, blood urea nitrogen.

Most of the participants in this study were independent community-dwelling older adults with higher functional status (mean Karnofsky Performance Scale was 89.4). They did not have impaired cognitive function, and the average Short Portable Status Questionnaire (SPMSQ) score was 0.26, less than 1 fault score. Cognitive impairment and mood disorders were more common in fallers as screened by SPMSQ and BSRS-5, respectively.

Compared with non-fallers, fallers had larger waist circumference, poorer visual acuity and lower body height. Serum hemoglobin and albumin levels were significantly lower in fallers and fallers had higher creatinine levels.

The significant variables listed in Table 1 were entered into the logistic model. Variables that were statistically significant (p<0.05) in the univariate analysis were age, gender, Parkinsonism, dementia, arrhythmia, body height, waist circumference, vision acuity, Karnofsky score, serum hemoglobin and albumin level. These variables included in the multivariate analysis by applying a multiple logistic stepwise regression procedure to obtain waist circumference, age, gender, visual acuity, Karnofsky score, and serum albumin level that independently correlated with falls. Adjusted odds ratios, which estimate the independent contribution of each variable to the likelihood of falling, are shown in Table 2 for the variables left in the final model.

**Table 2:** Multivariate logistic regression analyses of associated factors for falls.

	Beta coefficient	Standard error	Odds ratios (95% CI)	p-value
Age	0.028	0.014	1.03 (1.00–1.06)	0.05
Gender	0.661	0.181	1.94 (1.36–2.76)	<0.001
Waist	0.026	0.009	1.03 (1.01–1.05)	0.003
Visual acuity	−1.094	0.416	0.34 (0.15–0.76)	0.009
Karnofsky scale	−0.067	0.024	0.94 (0.89–0.98)	0.006
Albumin	−0.998	0.368	0.37 (0.18–0.76)	0.007

All statistical tests were two-tailed, and a significance level of p = 0.05 or less was used.

Multivariate analysis showed that odds ratio for the risk of falling was 1.94 (95% CI 1.36–2.76) when the female gender group is compared with the male gender group (reference group). The adjusted odds ratios of age and waist circumference were 1.03 (95% CI 1.00–1.06) and 1.03 (95% CI 1.01–1.05) respectively, so falls were associated with older age and larger waist circumference. The adjusted odds ratios of visual acuity, Karnofsky scale, and serum albumin level were 0.34 (95% CI 0.15–0.76), 0.94 (95% CI 0.89–0.98), and 0.37 (95% CI 0.18–0.76) respectively. There was an inverse relationship between the Karnofsky Performance Scale and falls. The poorer visual acuity and lower serum albumin level were associated with falls.

## Discussion

The identification of associated factors for falls as assessed by annual geriatric health examinations is an important method for safe-guarding the health of the community-dwelling older people. According to our findings, the prevalence of falls during a single year was 22.7%, which was similar to the findings in previous studies in Taiwan, Singapore [4], Hong Kong [5], and mainland China [6]. It was lower than the reported annual falls rates (35%–40%) in community-dwelling older people in western countries [14]. The difference between Asian and western countries might be caused by some factors, such as culture, lifestyle, activities in the daily life, family structure and social system. Most of the older people in Taiwan are living with children or nearby. Older people in western countries are more independent in the daily living and with outdoor activities.

Age, gender, waist circumference, visual acuity, Karnofsky score, and serum albumin level were identified as the independent risk factors for falls in our study. Older age[15]–[16], female gender[16]–[17], and poorer visual acuity[18]–[19] were noted as risk factors for falling in previous studies. One study showed that falls were significantly associated with poor visual acuity and higher waist-to-hip ratio [19]. Waist circumference, the indicator of central obesity, is a novel associated factor for falls. The association between large waist circumference and falling was independent of age, gender, and co-morbid conditions in our study.

One study showed obese people may be at a higher risk of falling than lightweight individuals [20]. Our study showed that body weight and body mass index were not significant risk factors for falls. The result was different from the previous study. Larger waist circumference, however, was shown to be an independent risk factor for falls. This factor was scarcely reported as an independent factor for falls before. People with a larger waist circumference had more central obesity and a higher tendency to develop metabolic syndrome and an unstable center of gravity, which can induce falls. The displacement of body's center-of-gravity before falls result in more severe injuries [21]. The observations in the study of lateral balance emphasize that older people may be particularly vulnerable to lateral instability that might increase their risk of falling [22]; [23]. Further research needs to study the relationship between abdominal obesity and lateral instability to induce falls.

Larger waist circumference is a result of central obesity, and waist circumference is a reliable marker in older adults for assessing visceral fat [24]. One study showed that obesity is associated with the frailty syndrome and falls in older women in cross-sectional study [25]. The recent study also showed the association of frailty with a high waist circumference, even among underweight older people [26]. People with truncal obesity have a higher risk to be frail and tend to induce falls. Our survey indicates that the measurement of waist circumference during a geriatric health examination is mandatory to identify the associated factors for falls in the community-dwelling older people.

Co-morbidities are common in older patients and increase with age. Fallers have a higher prevalence of Parkinson's disease [27], dementia, and arrhythmia[28]. Some studies also showed cognitive impairment can increase the risk for falls [29]–[30]. Depression needs to be considered in fall-risk assessments, as well as in population-based prevention and interventional strategies [31].

Serum hemoglobin and albumin levels were significantly lower in fallers. One study suggests a potentially important link between anemia and the risk of falls among the community-dwelling older people [32]. This finding implies that anemia is a modifiable risk factor for falls. Our survey showed that lower albumin level is an independent risk factor for falls, a fact that was seldom reported in previous studies. Hypoalbuminemia is regarded as a marker of malnutrition, influenced by infections, liver and renal diseases [33]. People with malnutrition have higher risk of falls, and further study is needed to demonstrate the association between falls and nutritional status.

There were several limitations in our studies. First, this is a cross-sectional study, the causality between associated factors and falls cannot be determined. Future prospective design of the study will be needed. Second, most of the participants in our study were from annual health examinations, and the results of the study cannot be generalized to all the community-dwelling old people. Third, we use the self-reported recall about falling, and it could introduce recall bias since only the least severe falls would be identified. Depending on the time period of recall, 13% to 32% of those with confirmed falls did not recall falling during the specific period of time[34]. Despite these limitations, this study is still valuable for health care providers to take care of older people from the perspective of fall prevention.

These associated factors for falls, especially waist circumference and albumin level, among the community-dwelling older Taiwanese people in our study should be included in a future screening tool to identify risk for falls and help to formulate strategies for fall prevention [35].

As assessed by our annual geriatric health examinations, larger waist circumference and other factors, such as older age, female gender, poorer visual acuity, lower Karnofsky score, and lower serum albumin level, were the independent associated factors for falls in Taiwanese aging population. Waist circumference should be included as a novel risk factor for falls.
